# The emergence and spread of one Coxsackievirus A16 Genogroup D novel recombinant strain that caused a clustering HFMD outbreak in Shanghai, China, 2016

**DOI:** 10.1038/s41426-018-0134-x

**Published:** 2018-07-18

**Authors:** Jiayu Wang, Zheng Teng, Wei Chu, Fanghao Fang, Xiaoqing Cui, Xiaokui Guo, Xi Zhang, Bruce R. Thorley, Yongzhang Zhu

**Affiliations:** 1 Microbiology Laboratory, Shanghai Municipal Center for Disease Control and Prevention, Shanghai, China; 2 Microbiology Laboratory, Huangpu Center for Disease Control and Prevention, Shanghai, China; 3 Department of Microbiology and Immunology, Institutes of Medical Science, Shanghai Jiao Tong University School of Medicine, Shanghai, China; 4 National Enterovirus Reference Laboratory, WHO Polio Regional Reference Laboratory, Victorian Infectious Diseases Reference Laboratory, Doherty Institute, Melbourne, VIC 3000 Australia; 5 Department of Clinical Microbiology, Institute of Antibiotics, Huashan Hospital, Fudan University, Shanghai, China

Dear Editors,

We report the first outbreak and spread of one Coxsackievirus A16 (CV-A16) novel Genogroup D recombinant strain in Shanghai, China, in 2016, 2 years after an initial report in France^1^. CV-A16 belongs to the Enterovirus (EV) A species and is one of the major serotypes that causes hand, foot, and mouth disease (HFMD). CV-A16 can be classified into three genogroups, A–C, based on the VP4 and VP1 gene sequences^2–5^. Genogroup B can be further divided into B1 (B1a–B1c) and B2^6^. CV-A16 Genogroup D is a novel recombinant genogroup. The epidemiological origin can be traced to Peru^7^. Hassel et al. reported its first emergence and circulation in France during 2010–2014^1^ and provided further detailed evidence of an intertype recombinant origin of CV-A16 genogroup D. To the best of our knowledge, no more report of the novel genogroup D could be found in the PubMed database.

The genogroup D Shanghai strain, designated SH-HP-16-51, was isolated from a mild HFMD case collected by our laboratory surveillance system on 5 October 2016. The patient was a 3-year-old girl. The epidemiological origin was traced to an aggregated case of 14 suspected HFMD patients reported in Pudong CDC. Between 2 and 5 October, 11 and 3 students among these 14 patients belonged to two adjacent classes in one kindergarten, respectively. The first case occurred on 2 October with fever (high temperature 38.9 °C), sore throat, and oral ulcers, and the child was diagnosed with HFMD on the next day. Unfortunately, clinical samples of only two patients were successfully collected in this outbreak because the kindergarten was quickly closed before we could collect other patient samples. However, no EV RNA was detected in the first case sample. In addition, although the guardians of both the first and second cases denied any history of contact with foreigners or travel abroad 2 weeks before the onset, we cannot exclude the possibility that the SH-HP-16-51 strain originated from other geographic locations, like France, because of increasingly frequent international business and communication.

The EV genome of strain SH-HP-16-51 was detected in a throat swab after RNA extraction. Pan EV and CV-A16 were confirmed with a commercial real-time RT-PCR Kit (BioPerfectus Technologies, Jiangsu, China). A human rhabdomyosarcoma cell line was then used for virus isolation. When typical cytopathic effects occurred (data not shown), culture medium and cells were harvested for purification. The complete genome was determined as described^1,8^ and submitted to GenBank (accession number: MG948605). The SH-HP-16-51 genome was 7231 bp long. Phylogenetic trees were constructed by using nucleotide sequences of the VP1, P1, P2, and P3 regions of the reference strains representing all genogroups with complete genome sequences. Phylogenetic analyses based on the complete VP1 sequence (891 bp) provided evidence that the isolate SH-HP-16-51 belonged to CV-A16 genogroup D and was most closely related to the strains isolated from France and Peru (Fig. 1a). These strains constituted a lineage distinct from other CV-A16 sub-genogroups and were placed into the same clade D when constructing phylogenetic trees using the P1, P2, and P3 genome regions (Figure S1). Furthermore, phylogenetic analysis based on VP2–VP4, 2A–2C, 3A–3D gene sequences also showed similar results (data not shown).

**Fig. 1:**
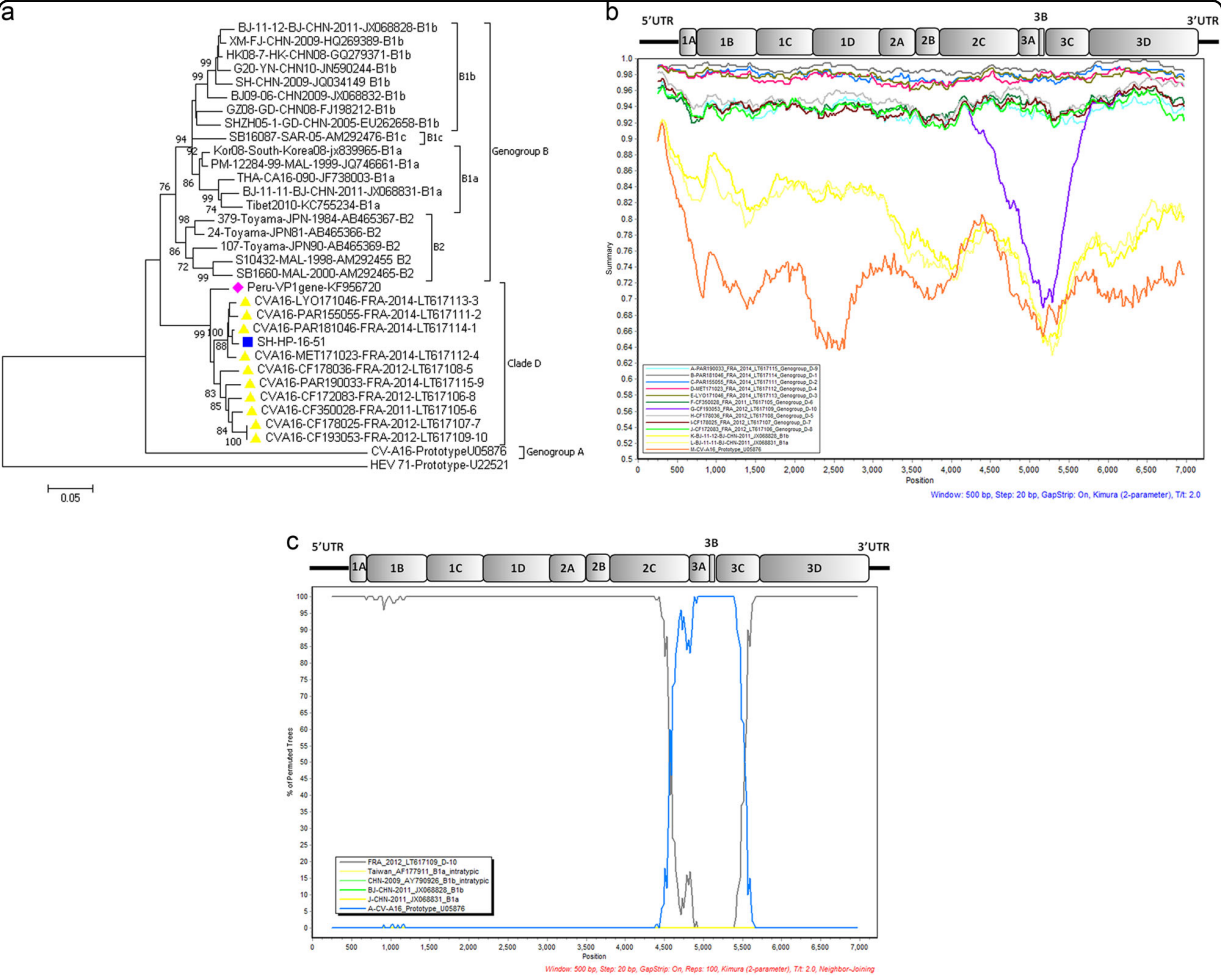
**a** Phylogenetic tree based on complete CV-A16 VP1 sequences. The tree was inferred with the neighbor-joining method from genetic distances calculated using the P distance algorithm. The tree topology was assessed with 1000 bootstraps. CV-A16 strain G10 (South Africa, 1951) was the only sample assigned to clade A. HEV 71 BrCr was used as an outgroup. **b** Nucleotide similarity between all genogroups of CV-16 full-length genomes. The similarity plots were obtained with a dataset of 14 CV-A16 genomes, including 10 clade D strains reported in France, the CV-A16 strain G10 and two clade B strains. The Shanghai isolate SH-HP-16-51 was used as the query sequence. G10, BJ/CHN/2011(JX068828) and BJ/CHN/2011(JX068831) were chosen as the representative strains of genotype A, B1a and B1b, respectively. A sliding window of 500 nucleotides in 20 nucleotide steps was used in this analysis. **c** Bootscan analyses of clades A, B and D of CV-A16 strains on the basis of full-length genomes. The Shanghai isolate SH-HP-16-51 was the query sequence. G10, FRA12(LT617109), BJ/CHN/2011(JX068828) and BJ/CHN/2011(JX068831) were chosen as the reference strains of A, D, B1a and B1b, respectively. AF177911 and AY790926 were chosen as the representative intratypic recombinant strains. The vertical axis indicates the percent nucleotide identity between the parental sequences. The horizontal axis shows the nucleotide positions of the nucleotide sequences

Nucleotide sequence identities between different CV-A16 genogroups and an outgroup (enterovirus 71 prototype) were compared (Table S1). Sequences of the capsid region (P1) of the SH-HP-16-51 strain showed 93.74–98.87% nt identity with the genogroup D strains reported in France, suggesting that the Shanghai strain belonged to the same genogroup. In nonstructural regions (P2 and P3), the Shanghai strain also had the highest sequence identity with the genogroup D strains, showing 90.88–98.44% and 90.41–98.89% nt identity, respectively. Comparative analysis of the ORF sequences also showed similar amino acid changes to those reported^1^ (data not shown) between clades B and D. Hence, the high nt identity between the SH-HP-16-51 strain and the French strains strongly indicated the potential common evolutionary origin. Moreover, we performed similarity plot and bootscan analyses using whole-genome sequences. The Shanghai isolate showed a greater similarity to the sequences of clade D sampled in France, with more than 93% nt identity (Fig. 1b), strongly supporting clustering between strain FRA12 (LT617109) and SH-HP-16-51 at nucleotide positions 300–4400, between CV-A16 prototype (U05876) and SH-HP-16-51 at nucleotide positions 4700–5400, and again between CV-A16 prototype (U05876) and SH-HP-16-51 at nt positions 5500 to the 3′ end of the genome. These findings indicate that intratypic recombination events might have occurred between nt positions 4400 and 5500 (corresponding to 2C-3C region) (Fig. 1c).

Seroprevalence of neutralizing antibody (NtAb) titers against one common CV-A16 clinically isolated strain (CV-A16_C_) and the SH-HP-16-51 strain were further conducted as previously described^9^. CV-A16_C_ (evolutionary branch: B1b; GenBank accession number: GQ429229) was isolated from a Shandong HFMD patient in 2007. One hundred serum samples from healthy children (including 58 males and 42 females) were collected in 2017. The overall seropositive rates (NtAb titer more than 8) against CV-A16_C_ and SH-HP-16-51 were 67% and 23%, respectively. Among these, 40% of healthy children had a high CV-A16_C_ NtAb titer (more than 16), while none of them had a high SH-HP-16-51 NtAb titer. Except for one case, all of these children with NtAb-positive SH-HP-16-51 had high CV-A16_C_ NtAb titer, indicating the common susceptibility of children to SH-HP-16-51 infection, as well as its potential circulation and outbreak in China in the future.

HFMD causes a substantial disease burden in the Asia-Pacific region, especially in children below 5 years of age. CV-A16 is one of the most common causative pathogens for HFMD. Recombination is a common phenomenon in EV evolution^10,11^. Our study is the first report of CV-A16 novel recombinant genogroup D in the Asia-Pacific region. Although different CV-A16 lineages have been identified in France and other countries, due to community transmission, they have seldom been associated with HFMD outbreaks. The Shanghai strain SH-HP-16-51 was isolated from one mild case in an HFMD outbreak. Whether the emergence of genogroup D will lead to large-scale transmission in mainland China remains unknown. However, no more CV-A16 genogroup D strains could be found in Shanghai by the end of 2017 (data not shown). Although the CV-A16 NtAb in the population induced low immune responses against the new CV-A16 recombinant genogroup, the low-titer NtAb did not produce effective protection against recombinant genogroup D or the onset of herpangina. Further studies emphasize the need for improving novel recombinant CV-A16 genogroup D surveillance. Genetic and antigenic characterization studies will no doubt assist in efforts to prevent and control potential transmission and epidemics.

## Electronic supplementary material

Figure S1

Table S1

Supplementary files
