# Supplementary figures and images for: The emergence and spread of one Coxsackievirus A16 Genogroup D novel recombinant strain that caused a clustering HFMD outbreak in Shanghai, China, 2016
Source: Emerg Microbes Infect. 2018 Jul 18;7:131. doi: 10.1038/s41426-018-0134-x (PMC6052075; doi:10.1038/s41426-018-0134-x)

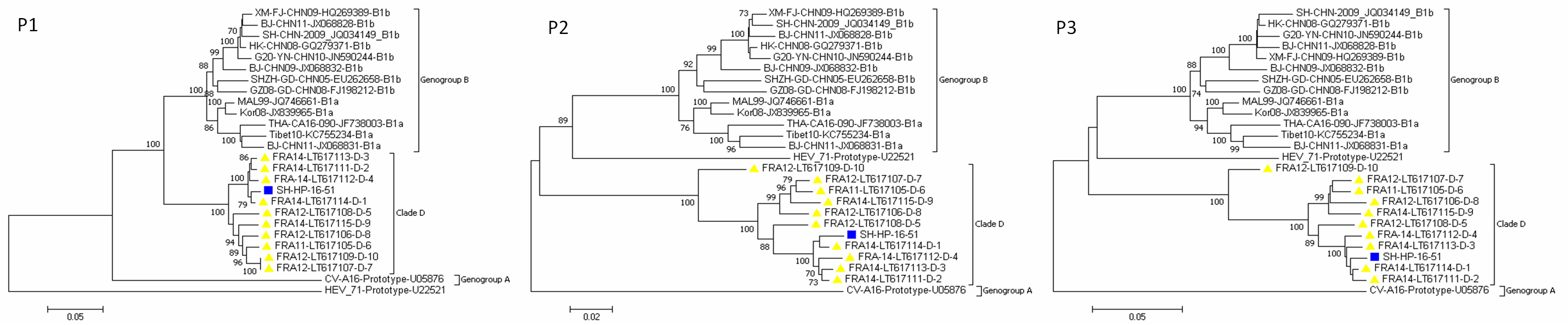

Supplement: Supplementary file 1 — Figure S1 [file 41426_2018_134_MOESM1_ESM.tif]
