# Supplementary material for: The emergence and spread of one Coxsackievirus A16 Genogroup D novel recombinant strain that caused a clustering HFMD outbreak in Shanghai, China, 2016
Source: Emerg Microbes Infect. 2018 Jul 18;7:131. doi: 10.1038/s41426-018-0134-x (PMC6052075; doi:10.1038/s41426-018-0134-x)
Supplement: Supplementary file 3 — Supplementary files [file 41426_2018_134_MOESM3_ESM.docx]

**Figure S1** Phylogenetic tree based on complete CV-A16 P1, P2 and P3 sequences. The tree was inferred with the neighbor-joining method from genetic distances calculated using the P distance algorithm. The tree topology was assessed with 1000 bootstraps. CV-A16 strain G10 (South Africa, 1951) was the only sample assigned to clade A. HEV 71 BrCr was used as an outgroup.

**Table S1**| Pairwise nucleotide sequence identities between SH-HP-16-51 genomic sequences and CV-A16 genogroup A, B and D strains. BJ/CHN/2011(JX068828), BJ/CHN/2011(JX068831) and FRA11(LT617105) were chosen as the representative strains of B1a, B1b and D.
